# Multi-Phase CT-Based Radiomics Nomogram for Discrimination Between Pancreatic Serous Cystic Neoplasm From Mucinous Cystic Neoplasm

**DOI:** 10.3389/fonc.2021.699812

**Published:** 2021-12-01

**Authors:** Jiahao Gao, Fang Han, Xiaoshuang Wang, Shaofeng Duan, Jiawen Zhang

**Affiliations:** ^1^ Department of Radiology, Huashan Hospital North, Fudan University, Shanghai, China; ^2^ Department of Radiology, Huashan Hospital, Fudan University, Shanghai, China; ^3^ Department of Life Sciences, GE Healthcare, Shanghai, China

**Keywords:** pancreatic cystic neoplasm, radiomics, nomogram, contrast-enhanced computed tomography (CECT), texture analysis

## Abstract

**Purpose:**

This study aimed to develop and verify a multi-phase (MP) computed tomography (CT)-based radiomics nomogram to differentiate pancreatic serous cystic neoplasms (SCNs) from mucinous cystic neoplasms (MCNs), and to compare the diagnostic efficacy of radiomics models for different phases of CT scans.

**Materials and Methods:**

A total of 170 patients who underwent surgical resection between January 2011 and December 2018, with pathologically confirmed pancreatic cystic neoplasms (SCN=115, MCN=55) were included in this single-center retrospective study. Radiomics features were extracted from plain scan (PS), arterial phase (AP), and venous phase (VP) CT scans. Algorithms were performed to identify the optimal features to build a radiomics signature (Radscore) for each phase. All features from these three phases were analyzed to develop the MP-Radscore. A combined model comprised the MP-Radscore and imaging features from which a nomogram was developed. The accuracy of the nomogram was evaluated using receiver operating characteristic (ROC) curves, calibration tests, and decision curve analysis.

**Results:**

For each scan phase, 1218 features were extracted, and the optimal ones were selected to construct the PS-Radscore (11 features), AP-Radscore (11 features), and VP-Radscore (12 features). The MP-Radscore (14 features) achieved better performance based on ROC curve analysis than any single phase did [area under the curve (AUC), training cohort: MP-Radscore 0.89, PS-Radscore 0.78, AP-Radscore 0.83, VP-Radscore 0.85; validation cohort: MP-Radscore 0.88, PS-Radscore 0.77, AP-Radscore 0.83, VP-Radscore 0.84]. The combination nomogram performance was excellent, surpassing those of all other nomograms in both the training cohort (AUC, 0.91) and validation cohort (AUC, 0.90). The nomogram also performed well in the calibration and decision curve analyses.

**Conclusions:**

Radiomics for arterial and venous single-phase models outperformed the plain scan model. The combination nomogram that incorporated the MP-Radscore, tumor location, and cystic number had the best discriminatory performance and showed excellent accuracy for differentiating SCN from MCN.

## Introduction

Pancreatic cystic neoplasms (PCNs) have been increasingly diagnosed in recent years as a direct result of the extensive use of abdominal cross-sectional imaging. The prevalence of incidentally discovered PCNs in the general population has been reported to range from 2.6 to 19.6% (1, 2). Considerable attention has been focused on serous cystic neoplasms (SCNs) and mucinous cystic neoplasms (MCNs) because of the significant difference in the probability of malignant transformation between the two (3). SCNs have an extremely low incidence of malignancy (4). The current management strategy for SCN is conservative, based on regular surveillance with rare interventions performed only because of symptoms (5, 6). MCNs are diagnosed almost exclusively in middle-aged women, but with a very definite potential for malignant transformation (7–9). In contrast to SCN, surgical resection has been advocated for many, if not most, MCN patients. Recognizing the marked difference in the risk of malignancy and the consequent nearly opposite clinical management strategies between these cystic neoplasms, it is vital to correctly discriminate between the two.

Currently, even the high-quality imaging modalities such as computed tomography (CT) and ultrasound do not provide adequate discrimination between SCN and MCN (10, 11). Clearly, radiological imaging approaches, especially multi-detector computed tomography (MDCT), play a pivotal role in the preoperative diagnosis of PCNs. It has been reported that the discrimination efficacy of CT for SCNs was ranged from 27 to 91% (12, 13). Compared with CT, MRI/magnetic resonance cholangiopancreatography (MRCP) could further improve the diagnostic accuracy of PCNs, with an accuracy of 40–95% (14, 15) providing a better view of the pancreatic duct system and allowing to detect the presence of a solid component or mural nodule. Endoscopic ultrasound with fine-needle aspiration (EUS-FNA) has become a promising tool for classifying specific subtypes of PCNs. Adding EUS-FNA to CT and MRI has improved the diagnostic accuracy by 36% and 54%, respectively (16). Balanced against these potential benefits is the invasive nature of EUS and the variable risk of FNA-associated complications (17). These limitations and the significant cost curtail their application for the routine evaluation of PCNs (18).

Radiomics is an emerging and rapidly developing method for advanced image analysis. Relative to PCNs, radiomics has been successfully applied to the entire spectrum of the disease process, including differential diagnosis, malignant assessment, and prognosis prediction (19–21). Most radiomics studies utilizing MDCT pancreatic scans have been limited to the venous phase (VP) for feature extraction. Clearly, the different phases reflect unique vascular enhancement and texture information. Logically, a radiomics model constructed from a plain scan (PS) or arterial phase (AP) scan would augment diagnostic efficiency. To the best of our knowledge, no prior studies have reported feature extraction of all three phases of contrast-enhanced CT to discriminate PCN subtypes.

Our aim was to compare the predictive efficacy of each single-phase radiomics model, and then to construct a combination nomogram, incorporating a multi-phase (MP) radiomics model with clinical imaging factors that would noninvasively and accurately discriminate SCNs from MCNs.

## Materials and Methods

### Patient Population

The Institutional Review Board of Huashan Hospital of Fudan University approved this retrospective study, and the requirement for informed consent was waived. Patients who were diagnosed with SCNs or MCNs for whom surgical resection was performed in our hospital between January 2011 and December were enrolled in this study. The inclusion criteria were: (1) SCNs or MCNs with surgical pathologic confirmation; (2) contrast-enhanced CT scans (slice thickness: 1.5 mm) performed within one month prior to pancreatic surgery. The exclusion criteria were: (1) CT images with serious artifacts and (2) patients whose radiomics features could not be successfully extracted from the CT images. The details of patient enrollment are shown in **Figure S1** in **Supplementary Materials**. The final study group comprised 115 patients with SCNs and 55 with MCNs. Patients were randomly grouped in a ratio of 7:3, with 120 and 50 patients in the training and validation cohorts, respectively.

**Figure 1:**
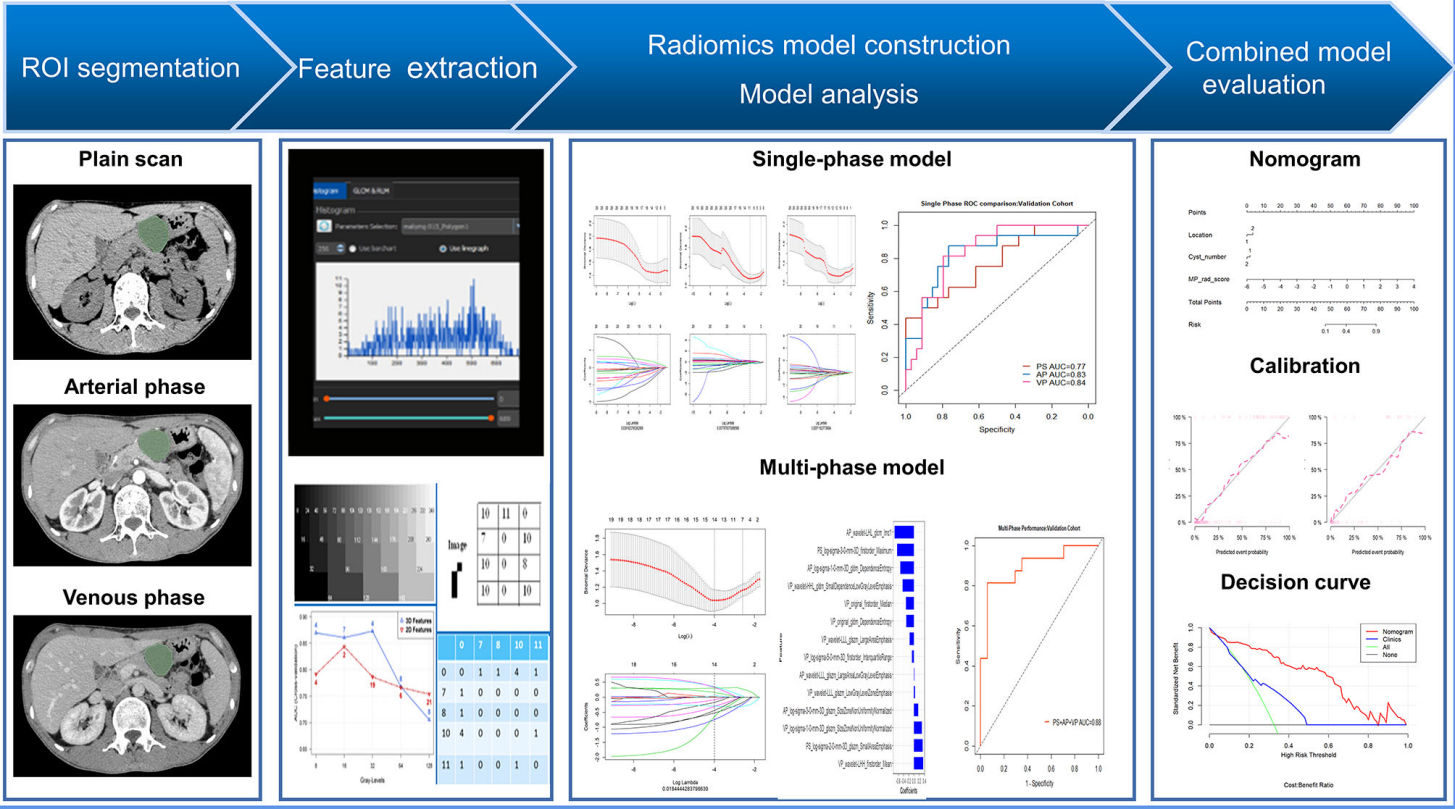
Radiomics workflow.

Patient demographic and clinical information was collected from the hospital medical record system. Demographic information (age and sex) and eight imaging factors known to be valuable in distinguishing SCNs from MCNs from previous studies were selected as the basis for constructing the clinical model (22, 23). Two radiologists with considerable experience in abdominal imaging (13 and 6 years, respectively) evaluated the features in consensus including: (1) lesion size, (2) tumor location (head, neck, body, and tail), (3) cyst number (single or multiple), (4) calcification (absent or present), (5) septation (absent or present), (6) lesion shape (oval or irregular lobulation), (7) wall enhancement (absent or present), and (8) mural nodules (absent or present). Both radiologists were blinded to the correlative pathological details. Among these, the lesion size was outlined and decided unanimously by two doctors simultaneously, while the other features were assessed by each doctor, and the results were derived separately. If the two radiologists did not agree on a specific feature in the same patient, a third expert with 23 years’ experience in abdominal radiology reviewed the features and helped establish the final decision. The inter-reader agreement of imaging factors was also assessed, as shown in **Supplementary Materials** (**Table S1**). The framework of the study is shown in **Figure 1**.

### Image Acquisition

CT examinations of all patients were performed using the same 256-slice CT system (Brilliance iCT, Philips Medical Systems, The Netherlands). All pancreatic CT images were acquired using a standard dual-phase scanning protocol. The CT scan parameters were as follows: 120 kV; 150–200 mAs; rotation time, 0.5-0.75 s; collimation, 128×0.625 mm; matrix, 512×512; and slice thickness, 1.5 mm. An anionic contrast agent (370 mgI/mL, Iopamidol-370, GEhealthcare, Princeton, NJ) was administered at a dose of 1.5 mL/kg, 3.0 mL/s. AP images were obtained 30 s after the injection of contrast agent, and VP images were obtained 45 s after the AP acquisition. All images were downloaded from the hospital archives.

### Tumor Segmentation and Single-Phase Radiomics Feature Extraction

PS, AP, and VP CT images in each patient were used for feature extraction. The window width and window level were 300 and 40 HU, respectively. For each phase, one radiologist (13 years’ experience in abdominal imaging) segmented the lesion contour on each slice using open-source software (3D Slicer version 4.11.0; Boston, MA). With the technical support of a radiomics software based on Python (Pyradiomics version 3.0.0; https://github.com/Radiomics/pyradiomics) (24), radiomics features were extracted in three-dimensional volume for each phase. The extracted features were classified into six categories: (1) shape features, (2) first order statistics, (3) gray level co-occurrence matrix features, (4) gray-level run length matrix features, (5) gray-level size zone matrix features, and (6) gray-level dependence matrix features. Details of the features are provided in **Supplementary Materials I**.

To estimate both intra- and inter-observer reproducibility of extracted features, 60 patients were randomly chosen for a repeat region of interest (ROI) segmentation at 30 days following the initial segmentation, performed by the same radiologist and an additional one (with 6 years’ experience in abdominal imaging). The radiologists were blinded to the associated clinical and pathological information. The intra- and inter-class correlation coefficients (ICCs) were used to evaluate feature reliability (25).

### Feature Selection, Single-Phase Radiomics Signature Construction, and Performance Comparison

**Figure 2:**
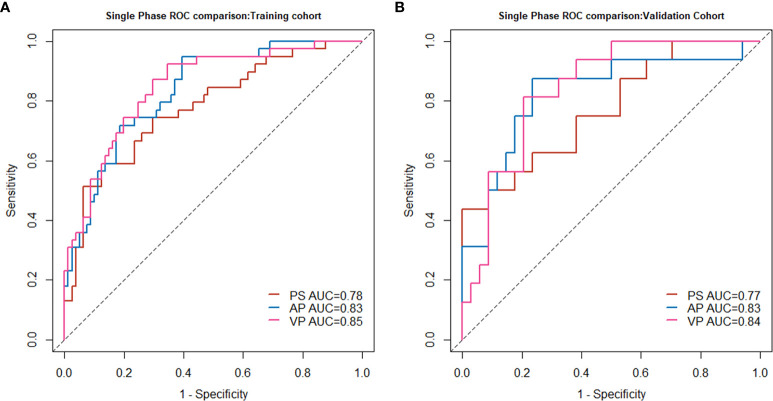
Single-phase radiomics model performance in the training cohort **(A)** and validation cohort **(B)**.

In the training cohort, a three-step procedure was developed to select the radiomics features extracted in each phase. First, features with both intra- and inter-ICC less than 0.75, were excluded from this process. The mRMR method and the least absolute shrinkage and selection operator (LASSO) algorithm were used to select the most robust and optimal features to construct the single-phase radiomics model. The selected optimal features were then combined with its coefficient in the LASSO regression to construct the radiomics signature: Radscores (including PS-Radscore, AP-Radscore, VP-Radscore). The Mann-Whitney U test was used to evaluate the discrimination capability of the each-phase Radscore. We also used receiver operating characteristic (ROC) curve analysis and area under the curve (AUC) values to compare the performance of the single-phase radiomics signature. The detailed performance of each of the radiomics signatures is shown in **Figure 2** and **Supplementary Materials II**. We also constructed and evaluated the two-phase combined radiomics model (**Supplementary Materials III**).

### Combined Model Building and Nomogram Development

A MP radiomics feature set was developed by integrating all 3654 (1218*3) features of the three phases. We then used the same three-step feature extraction method to obtain an MP radiomics signature, MP-Radscore. The discrimination capability of the MP radiomics model was also evaluated using the Mann-Whitney U test and ROC curve analysis. Univariate analysis was conducted to estimate the differences between SCN and MCN patients for each clinical and imaging feature. In the training cohort, variables with *P* < 0.100, in the univariate regression, were then allocated to a multivariable logistic regression. The clinical model was constructed by incorporating factors with *P* < 0.100 in the multivariate analysis (26). Finally, a combination multivariate logistic model was constructed using MP-Radscores together with selected clinical imaging factors. Variance inflation factor (VIF) analysis was performed on the combination model to further reduce the probability of overfitting. The nomogram was developed to visualize the optimal model, specifically to score each patient and quantify the degree of disease tendency.

### Model Validation and Clinical Use Evaluation

The combination model was first evaluated in the training cohort (n = 120) and subsequently confirmed in the validation cohort (n = 50). ROC curves and AUC values were used to evaluate the discriminatory performance of the combined models. Calibration curves and the Hosmer-Lemeshow test were conducted to estimate the consistency between the predictive results of the combination model and expected probabilities. We also used the Delong test to compare the predictive efficiency between the combination model and the venous radiomics approach to confirm the advances of our combination model.

Decision curve analysis (DCA) was performed to determine the clinical value of the nomogram and calculate the net benefits of the models at different threshold probabilities (27).

### Statistical Analysis

Continuous variables are presented as means and standard deviations. Student’s t-test and chi-square test were employed to evaluate the statistical differences in continuous and discrete variables, respectively. In the ROC test, accuracy, sensitivity, and specificity at the cutoff value were calculated to evaluate the efficiency of the radiomics model, clinical model, and the combination model. The inter-reader agreement of imaging factors was assessed using the kappa test, and the simple kappa coefficient was used as an assessment criterion for consistency. A two-tailed *P* value less than 0.05, was deemed as statistically significant. All statistical analyses were performed using the R software (version 3.6.3). The R packages and the main code used are included in **Supplementary Materials V**.

## Results

### Patients Characteristics

Patient characteristics are summarized in **Table 1**. There were 120 patients (SCN: 81, MCN: 39) in the training cohort and 50 (SCN: 34, MCN: 16) in the validation cohort. No significant differences were found in the clinical and imaging features between the two cohorts.

**Table 1:** Characteristics of Patients in the Training and Validation Cohorts.

Characteristics	Training Cohort (n = 120)			Validation Cohort (n = 50)		
	SCN (n = 81)	MCN (n=39)	*P*	SCN (n = 34)	MCN (n = 16)	*P*
**Age, mean ± SD**	51.2 ± 11.5	50.8 ± 14.9	.890	56.4 ± 14	53.9 ± 11.1	.523
**Sex, No (%)**						
Male	15 (18.5)	3 (7.7)	.199	11 (36.7)	2 (12.5)	.251
Female	66 (81.5)	36 (92.3)		23 (63.3)	14 (87.5)	
**Lesion size (cm)**	4.0 ± 2.5	4.7 ± 2.1	.113	4.3 ± 2.4	5.5 ± 2.7	.105
**Tumor location**						
Head and neck	37 (45.7)	9 (23.1)	.029	13 (38.2)	13 (81.2)	.011
Body and tail	44 (54.3)	30 (76.9)		21 (61.8)	3 (18.8)	
**Cyst number**						
Single	50 (61.7)	33 (93.9)	.019	16 (47.1)	12 (75.0)	.120
Multiple	31 (38.3)	6 (6.1)		18 (52.9)	4 (25.0)	
**Calcification**						
Absent	53 (65.4)	25 (64.1)	1.00	20 (58.8)	12 (75.0)	.426
Present	28 (34.6)	14 (35.9)		14 (41.2)	4 (25.0)	
**Septation**						
Absent	40 (49.4)	14 (35.9)	.232	11 (32.4)	8 (50.0)	.375
Present	41 (50.6)	25 (64.1)		23 (67.6)	8 (50.0)	
**Lesion shape**						
Oval	50 (61.7)	27 (69.2)	.548	20 (58.8)	12 (75.0)	.426
Irregular lobulation	31 (38.3)	12 (30.8)		14 (41.2)	4 (25.0)	
**Wall enhancement**						
Absent	57 (70.4)	26 (64.1)	1.00	23 (67.6)	11 (68.8)	1.00
Present	24 (29.6)	13 (35.9)		11 (32.4)	5 (31.2)	
**Mural nodules**						
Absent	64 (79.0)	35 (89.7)	.233	23 (67.6)	15 (93.8)	.096
Present	17 (21.0)	4 (10.3)		11 (32.4)	1 (6.2)	

### Feature Selection, Single-Phase Radiomics Signature Construction, and Performance Comparison

From each phase of contrast-enhanced CT scans, 1218 features were extracted. After ICC assessment applying the minimum criteria for intra- or inter-ICC values, 0.75, 765, 922, and 840 features remained from the PS, AP, and VP CT images, respectively. Of these, 30 features from each phase were retained using the mRMR algorithm for LASSO regression. Finally, 11,11, and 12 radiomics features were selected to construct the radiomics signature for the radiomics model of PS, AP, and VP CT scans, respectively. Features were identified using multivariate logistic regression analysis to construct single-phase radiomics signatures (PS-Radscore, VP-Radscore, and AP-Radscore). The formulas for calculating the Radscores and features selected for the single-phase radiomics model are presented in **Supplementary Materials II**.

In all three single-phase Radscores, there was a significant difference between SCN and MCN patients in the training cohort (*P* < 0.010), and importantly, this was confirmed in the independent validation cohort (*P* < 0.010). The PS, AP, and VP radiomics models yielded AUC values of 0.78, 0.83, and 0.85, respectively, for the training cohort, and 0.77, 0.83, and 0.84, respectively, for the validation cohort. The AUC values in the radiomics model in AP and VP were similar and higher than those in the radiomics model of the plain scan. The performance of the single-phase radiomics model is shown in **Figure 2**.

### Combined Model Building and Nomogram Development

**Figure 3:**
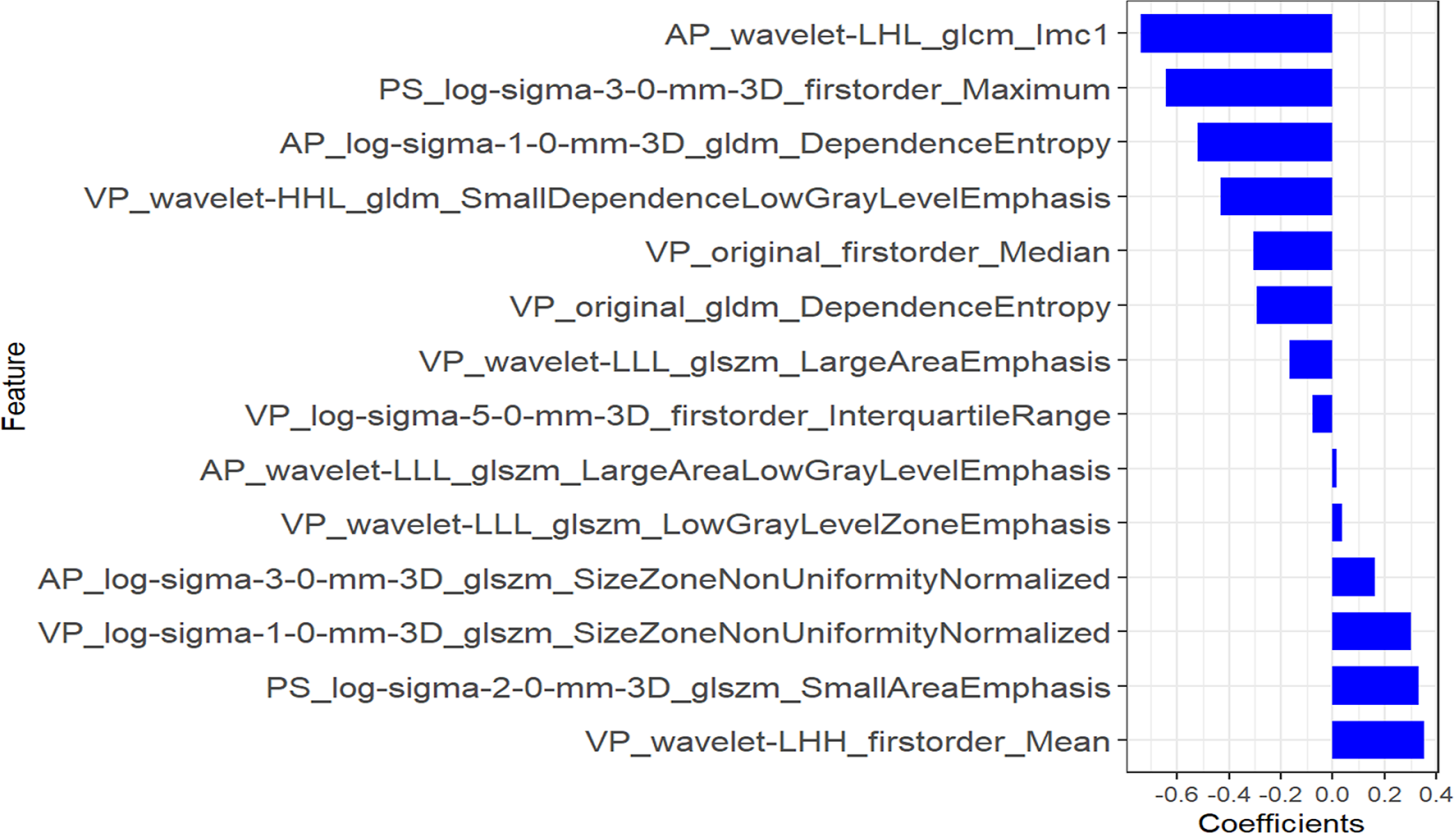
Features selected for multi-phase radiomics model construction.

**Figure 4:**
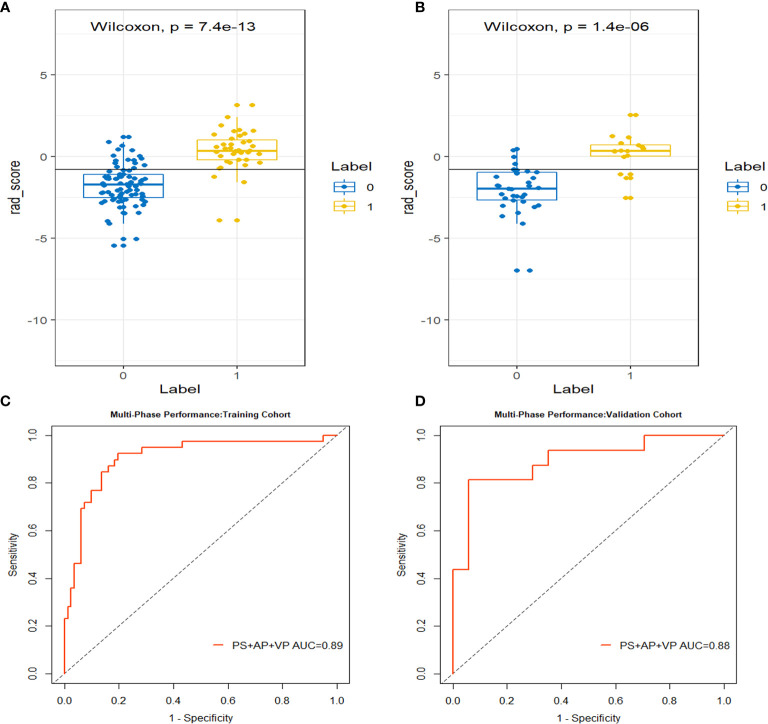
The box-dot plots of the multi-phase radiomics model in the training cohort **(A)** and the validation cohort **(B)**. The orange markers indicate patients with MCN while the blue markers indicate patients with SCN. The ROC curves for the multi-phase radiomics model in the training cohort **(C)** and the validation cohort **(D)**.

Using the three-step selection process described above for single-phase radiomics model construction, 14 features (including 2 in PS, 4 in AP, and 8 in VP) were similarly selected from the MP radiomics feature set (**Figure 3**). The MP-Radscore was built to improve the discrimination efficacy of the MP radiomics model. ROC curves showed that the MP radiomics model performed better than the models based on single CT phase (AUC: 0.89 and 0.88 in the training and validation cohorts, respectively). The performance of the MP radiomics model in the Mann-Whitney U test and ROC curves are shown in **Figure 4**. The detailed calculation formulas of the MP-Radscore and combined nomogram are included in **Supplementary Materials IV**.

**Figure 5:**
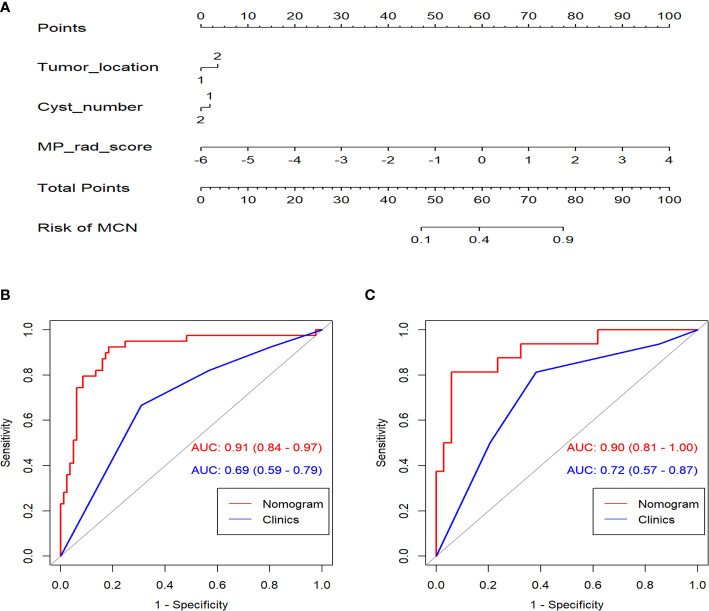
**(A)** The nomogram established for the combined model. ROC curves comparison between the nomogram and clinical model in the training **(B)** and validation cohorts **(C)**.

In the univariate analysis of the clinical model building, only tumor location and cyst number were significantly correlated with pathologic results (*P* < 0.100). Tumor location and cyst number were statistically significant (*P* < 0.100) in the multivariate logistic regression analysis, therefore comprising the clinical model. The results of the univariate and multivariate logistic regression analyses are shown in **Table 2**. The combination model was constructed by incorporating the MP-Radscore, tumor location, and cyst number. A nomogram was established to visualize the combined model **(** **Figure 5A** **).**

**Table 2:** Variables Elected for Combined Model and Clinical Model.

Intercept & Variable	Combined Model (95%CI)		Clinical Model (95%CI)	
	Odds Ratio	P Value	Odds Ratio	P Value
Intercept	0.74 (0.05,10.13)	<0.01	0.45 (0.06, 3.11)	<0.01
Tumor location	1.69 (0.54, 5.42)	0.05	2.65 (1.62, 2.70)	0.03
Cyst number	0.76 (0.23, 2.48)	0.01	0.30 (0.11, 0.75)	<0.01
MP-Radscore	4.25 (2.58, 7.96)	<0.01	NA	NA

NA, not available.

### Combination Model Validation and Clinical Use Evaluation

The combination nomogram exhibited best predictive performance (AUC: 0.91 and 0.90 in the training and validation cohorts, respectively) for the discrimination between SCNs and MCNs (**Figures 5B, C** and **Table 3**). The Delong test demonstrated statistical differences in AUC values between the combination nomogram and the clinical model (*P* < 0.010). Significant differences were also found in the ROC curves between the combination nomogram and VP model (Z = 1.962, *P* = 0.0497 < 0.0500) in the validation cohort. Calibration curves (**Figure S6**) revealed good agreement between the predictive and observation probabilities of our combination nomogram (*P* = 0.480 and 0.582 for the training and validation cohorts, respectively).

**Table 3:** Diagnostic performance of models in the training and validation cohorts.

Models	Training Cohort (n = 120)				Validation Cohort (n = 50)			
	Sensitivity	Specifity	Accuracy (95%CI)	AUC (95%CI)	Sensitivity	Specifity	Accuracy (95%CI)	AUC (95%CI)
**Clinical model**	0.67	0.69	0.68 (0.59, 0.76)	0.69 (0.59, 0.79)	0.50	0.79	0.70 (0.55, 0.82)	0.72 (0.57, 0.87)
**MP-Radscore**	0.91	0.80	0.84 (0.76, 0.90)	0.89 (0.82, 0.95)	0.81	0.88	0.82 (0.73, 0.89)	0.88 (0.77, 0.98)
**Combined** **nomogram**	0.92	0.81	0.85 (0.77, 0.91)	0.91 (0.84, 0.97)	0.71	0.90	0.78 (0.64, 0.88)	0.90 (0.81, 1.00)

**Figure 6:**
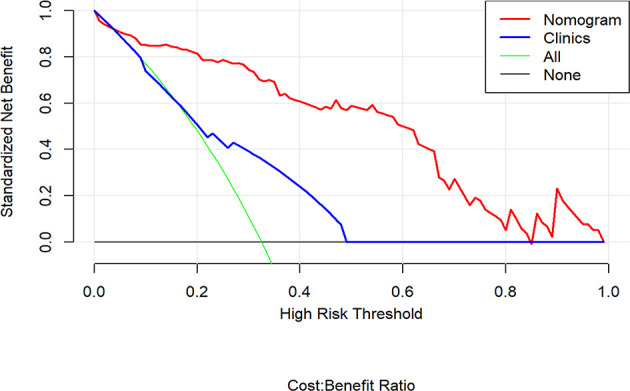
Decision curve analysis for the nomogram compared with clinical model in the validation cohort. It can be concluded that when the threshold probability is over 10% approximately, the nomogram could provide extra profit over the “treat-all” or “treat-none” scheme and the clinical model.

The decision curve analysis indicated that the combination nomogram provided a net benefit over either a “treat-all” or “treat-none” strategy, and the clinical model at a threshold probability over 10% (**Figure 6**). The combination nomogram demonstrated excellent clinical practicality.

## Discussion

In this retrospective study, we constructed and validated an MP CT-based radiomics nomogram to differentiate SCN from MCN. The combination model, incorporating the MP radiomics model plus clinical imaging factors, exhibited better diagnostic performance than any of the single-phase radiomics models or a clinical model alone did. The decision curve analysis also confirmed that the combination model achieved better discriminatory accuracy than the clinical model did. Relating specifically to the single-phase performance comparison, the radiomics model of the AP and the VP performed better than the PS model in terms of AUC values.

The exact morphologic details of MDCT are crucial to exclude tumor invasion of PCNs. Key imaging morphologic factors (tumor size, location, lesion shape, calcification, segmentation, etc.) derived from pathologic characteristics form the basis for radiologic differentiation of PCN subtypes (28, 29). Nevertheless, the diagnostic accuracy of cross-sectional imaging, such as MDCT, still falls short of ideal discrimination (30). Therefore, intrusive methods, such as EUS-FNA, have been developed to add diagnostic precision for preoperative PCN subtyping. Clearly, achieving this degree of accuracy requires highly skilled endoscopists and cytologists (31, 32).

Apart from the invasive techniques described above, radiomics offers a promising noninvasive technology intended to achieve similar results. We successfully established a combination radiomics model and achieved superior capacity to differentiate SCNs from MCNs. Among numerous clinical imaging factors, only cyst number and tumor location were statistically essential to be included in the combination model. Considering that the clinical and imaging features of PCNs pathologically diagnosed as SCN in the study were not consistent with typical SCN manifestations, we also analyzed and modeled these features. These results are consistent with a number of previous CT imaging studies. The differences in morphology that discriminate between SCNs and MCNs are limited to tumor location, lobular contour, and a large number of cysts (30, 33). The prediction accuracy of the clinical model alone was poor, with AUC values of only 0.69 and 0.63 in the training group and validation group, respectively. Even with high-quality CT scans interpreted by skilled radiologists, the accuracy of PCN subclassification remains disappointing.

A recent study assessed the discriminatory efficacy of conventional CT imaging features in distinguishing SCN from MCN and presented it by building a nomogram based on multivariate logistic regression (34). In contrast, our study not only considered conventional clinic-radiological features, but also incorporated radiomics features that reflected the deeper dimensional information of the images to construct a comprehensive model. The results showed that the combined model demonstrated better predictive ability than the clinical model alone. Several previous studies have applied radiomics to the differentiation of PCNs and have achieved good results (35, 36). However, further validation is required because of the limited amount of data (N < 80) and the high risk of overfitting. Moreover, a nomogram has not yet been established to visualize the radiomics model. Finally, they performed feature extraction almost uniquely on VP CT images. In the present study, in addition to VP CT scans, we also investigated the radiomics signatures on plain and AP CT images. The VP-Radscore AUC value was the best among the three single-phase radiomics models; similarly, the radiomics models of both the VP and AP had superior AUC values compared to those of the PS model. These results require verification, because they rely heavily on the experience of the radiologist performing manual segmentation (22). Interestingly, several radiomics studies have constructed radiomics models from only PS and have achieved good results in disease prediction (37, 38). The feature composition of the MP radiomics model included 8 features (57.1%) in the VP, 4 in the AP (28.6%), and only 2 (14.3%) in the PS. Our highest quantitative ranking of the VP is consistent with most previously published pancreatic radiomics research, while the PS was used less frequently. Therefore, this study is also significant in that it provides preliminary insight into the effect of contrast-enhanced CT scan phase on the predictive efficacy of imaging histology models and establishes a more comprehensive model to summarize various types of risk factors for prediction.

Our study has some limitations. First, this was a retrospective study that was conducted in a single center with a relatively small sample size. Large-scale external validation is needed to further demonstrate the clinical efficacy of the nomogram constructed here. Second, while our method of manual segmentation set the basis for excellent results, this was possible because of our relatively small number of cases. For widespread application of this technique, more research employing automatic or semi-automatic image segmentation is likely to be necessary. Third, the patients included in this study all had SCN or MCN confirmed using surgical pathology, and there may have been a selection bias. IPMN or other pancreatic cystic diseases need to be further studied to broaden the clinical application of this algorithm.

In conclusion, our study has established a novel multi-phase CT-based radiomics nomogram for a noninvasive preoperative differentiation of SCNs from MCNs. The nomogram could provide a reference basis for an accurate diagnosis, thereby avoiding unnecessary surgical resection in clinical practice. We also preliminarily explored the influence of specific feature extraction phases on the predictive efficacy of the radiomics model; the results may be enlightening to subsequent radiomics studies.

## Data Availability Statement

The raw data supporting the conclusions of this article will be made available by the authors, without undue reservation.

## Ethics Statement

The studies involving human participants were reviewed and approved by the Institutional Review Board of Huashan Hospital, Fudan University. Written informed consent for participation was not required for this study in accordance with the national legislation and the institutional requirements.

## Author Contributions

JG and FH: made the same effort in study, designed and carried out the experiments. XW: collected and sorted the data. SD: technology support. JZ: guided and modified the manuscript. All authors contributed to the article and approved the submitted version.

## Funding

The study was funded by National Key Research and Development Project in China (No. 2017YFC0113405), Launching Fund of Huashan Hospital (North Hospital) Affiliated to Fudan University (No. HSBY2020003).

## Conflict of Interest

Author SD was employed by GE Healthcare.

The remaining authors declare that the research was conducted in the absence of any commercial or financial relationships that could be construed as a potential conflict of interest.

## Publisher’s Note

All claims expressed in this article are solely those of the authors and do not necessarily represent those of their affiliated organizations, or those of the publisher, the editors and the reviewers. Any product that may be evaluated in this article, or claim that may be made by its manufacturer, is not guaranteed or endorsed by the publisher.
