# Supplementary material for: Multi-Phase CT-Based Radiomics Nomogram for Discrimination Between Pancreatic Serous Cystic Neoplasm From Mucinous Cystic Neoplasm
Source: Front Oncol. 2021 Dec 1;11:699812. doi: 10.3389/fonc.2021.699812 (PMC8672034; doi:10.3389/fonc.2021.699812)
Supplement: Supplementary file 1 [file DataSheet_1.doc]

**Supplementary Material**

**I. Radiomics features extraction methodology**

**II.** **Single-phase radiomics model construction and performance evaluation**

**III.** **Two-phase radiomics model construction and performance evaluation**

**IV. Multi-phase radiomics model construction and** **calculation formula of the MP-Radscore and combined nomogram.**

**V. R packages and code we used in this study**

**Fig. S1.** Recruitment pathway for patients in this study.

**Fig. S2.** The LASSO regression process of each phase radiomics signature building.

**Fig. S3.** Radiomics features selected for each phase’ signature building.

**Fig. S4.** Comparison of discrimination ability of single phase radiomics model .

**Fig. S5.** The comparison of two-phase radiomics models in ROC curves in the training cohort (A) and validation cohort (B).

**Fig. S6.** The calibration curves presented good consistency between the predictive and observation probabilities of the combined nomogram in the training cohort (A) and validation cohort (B).

**Table S1：**Inter-reader agreement assessment of the imaging factors

**I. R****adiomic****s features extraction methodology**

1218 radiomics features were extracted from each phase of the contrast-enhanced CT scans (including plain scan, arterial phase and venous phase). Finally, all 3654 features of the three phases constitute a multi-phase feature set. The radiomics features can be divided into six categories:(1) Shape features (n=14); (2) First Order Statistics (n=18); (3) Gray Level Cooccurrence Matrix (GLCM) features (n=22); (4) Gray Level Run Length Matrix (GLRLM) features (n=16); (5) Grey Level Size Zone Matrix (GLSZM) features (n=16); (6) Gray Level Dependence Matrix (GLDM) features (n=14). After 5 times of wavelet filter transform and 8 times of Laplacian of Gaussian (LoG) transform, all kinds of features except shape features increased by 14 times (1+5+8). Therefore, the total number of features extracted for each phase are:14*1(shape features)+(18+22+16+16+14)*14=1218.

The specific contents of each type of features could be seen on the website: <https://pyradiomics.readthedocs.io/en/latest/features.html.> Then we performed the minimum redundancy maximum relevance (mRMR) algorithm to further select the features. 30 characteristics that were most correlated with the results and least correlated with each other were selected for the LASSO regression. Finally, the radiomics signatures was constructed by the LASSO logistic regression model. The radiomics signature (Radscore) were composed of the features with non-zero coefficients in the LASSO regression, through a linear combination of their weighted coefficients.

**Fig. S1.** Recruitment pathway for patients in this study

195 patients with SCN or MCN were collected from January 2011 and December 2018 met the inclusion criteria.

Finally,170 patients were enrolled in this study.

21 patients whose CT images with serious artifacts.

4 patients whose radiomics features could not be successfully extracted from their CT images.


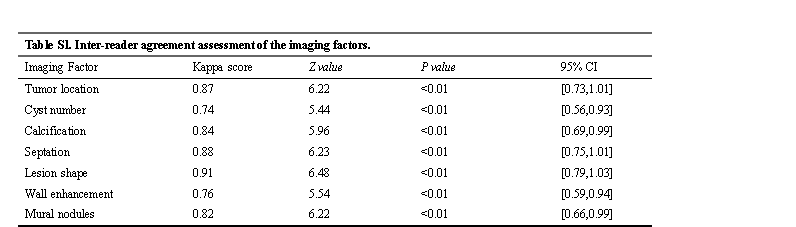


**II.** **Single-phase radiomics model construction and performance evaluation**

The specific calculation formulas of each phase Radscore and manifestations of the three phase radiomics models are shown below.

1. The calculation formula of Radscores in each phase radiomics model.

Plain scan radiomics model:

***PS-Radscore***=-0.17*PS_log-sigma-5-0-mm-3D_firstorder_Maximum +0.243*PS_original_shape_Sphericity +0.006*PS_original_firstorder_Kurtosis +0.132*PS_original_glrlm_LongRunLowGrayLevelEmphasis -0.02*PS_log-sigma-2-0-mm-3D_firstorder_Maximum -0.272*PS_log-sigma-4-0-mm-3D_firstorder_90Percentile +0.044*PS_wavelet-HLH_gldm_LargeDependenceEmphasis -0.024*PS_wavelet-HHL_firstorder_RootMeanSquared -0.05*PS_log-sigma-2-0-mm-3D_firstorder_Skewness -0.063*PS_original_firstorder_Median -0.371*PS_log-sigma-2-0-mm-3D_firstorder_RobustMeanAbsoluteDeviation -0.782

**Arterial phase radiomics model**：

***AP-Radscore*** = -0.277*AP_log-sigma-1-0-mm- 3D_gldm_DependenceEntropy -0.101*AP_wavelet-LLL_glcm_Autocorrelation- 0.269*AP_original_firstorder_90Percentile +0.254*AP_log-sigma-3-0-mm-3D_glszm_SizeZoneNonUniformityNormalized +0.235*AP_wavelet-HLL_firstorder_10Percentile +0.029*AP_log-sigma-2-0-mm-3D_glszm_SizeZoneNonUniformityNormalized -0.161*AP_original_gldm_DependenceEntropy +0.085*AP_log-sigma-2-0-mm-3D_firstorder_10Percentile -0.115*AP_wavelet-HLH_glszm_GrayLevelNonUniformity -0.191*AP_original_shape_Elongation +0.137*AP_log-sigma-1-0-mm-3D_glszm_SizeZoneNonUniformityNormalized -0.896

**Venous phase radiomics model**：

***VP-Radscore*** = -0.403*VP_original_firstorder_Median +0.369*VP_original_glrlm_LongRunLowGrayLevelEmphasis +0.01*VP_log-sigma-3-0-mm-3D_glszm_SizeZoneNonUniformityNormalized +0.159*VP_log-sigma-1-0-mm-3D_glszm_SizeZoneNonUniformityNormalized +0.311*VP_wavelet-LHH_firstorder_Mean -0.144*VP_wavelet-LLL_glszm_LargeAreaEmphasis +0.049*VP_wavelet-LLL_firstorder_Uniformity +0.309*VP_wavelet-LLH_glszm_SizeZoneNonUniformityNormalized +0.011*VP_wavelet-HHL_glszm_LargeAreaLowGrayLevelEmphasis -0.27*VP_wavelet-HHL_firstorder_RootMeanSquared -0.213*VP_log-sigma-3-0-mm-3D_gldm_DependenceVariance -0.197*VP_wavelet-LLL_glcm_JointAverage -0.899

1. The figures for single-phase performance comparison of radiomics model based on different phases.

**Fig. S2.** The LASSO regression process of each phase radiomics signature building.


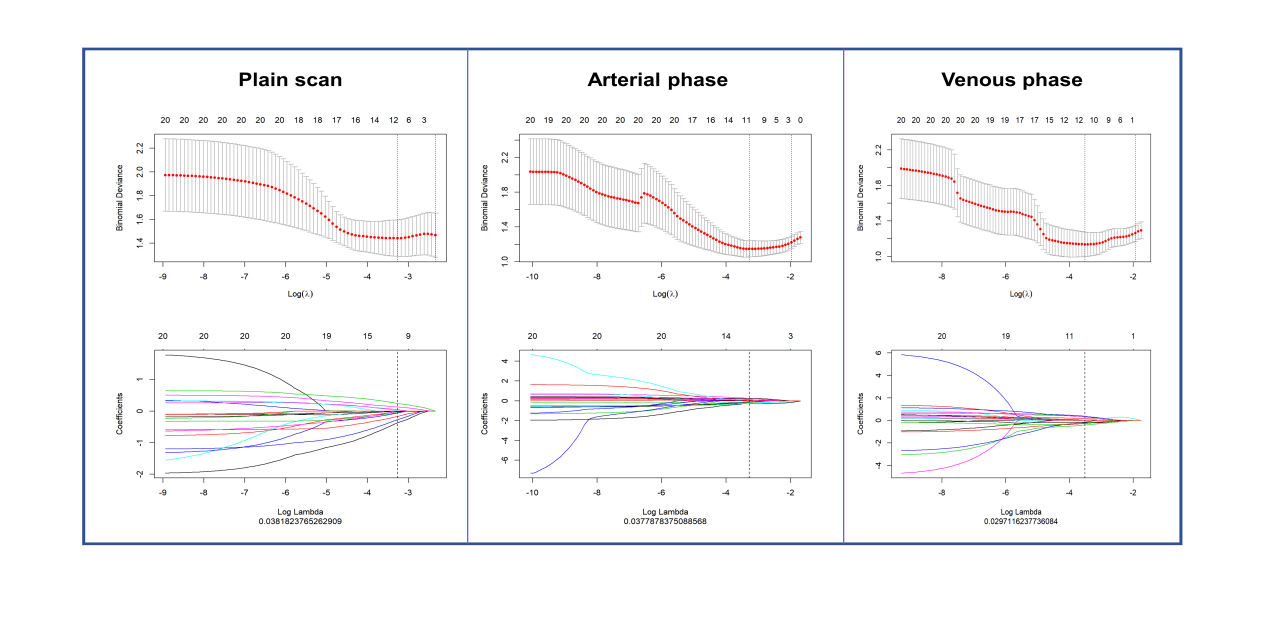


**Fig. S3.** Radiomics features selected for each phase signature building.


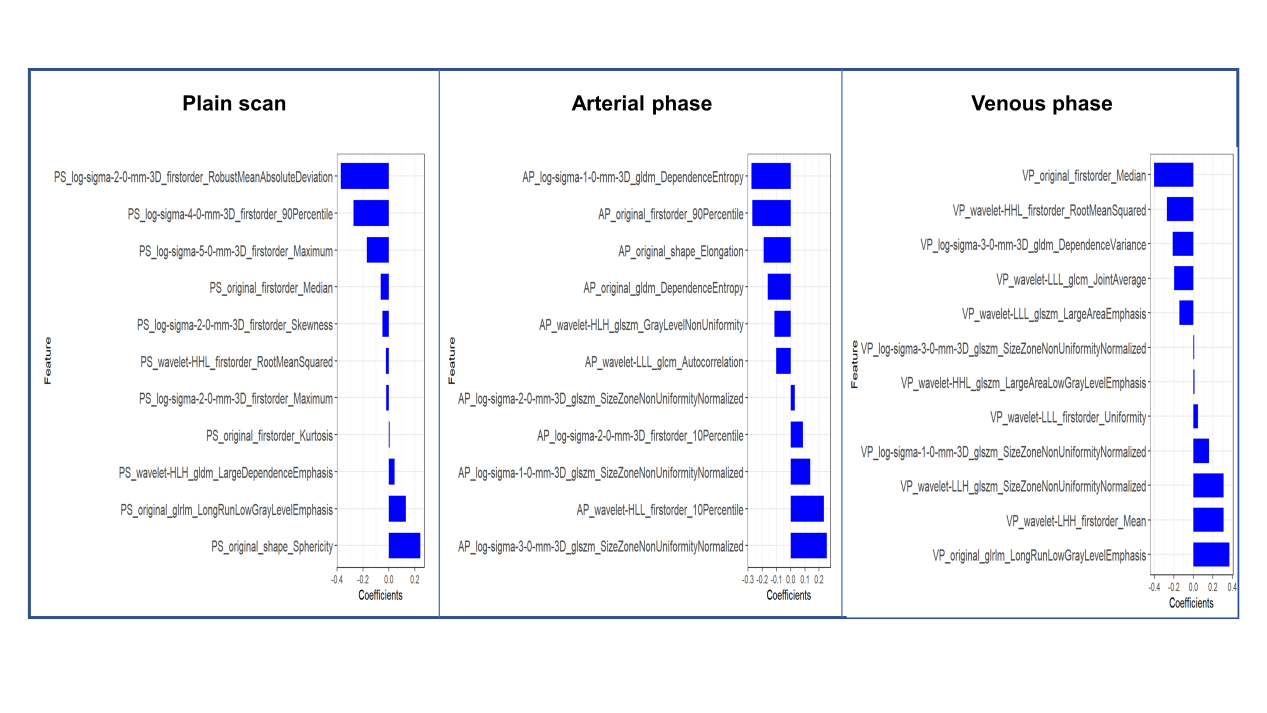


**Fig. S4.** Comparison of discrimination ability of single phase radiomics model .


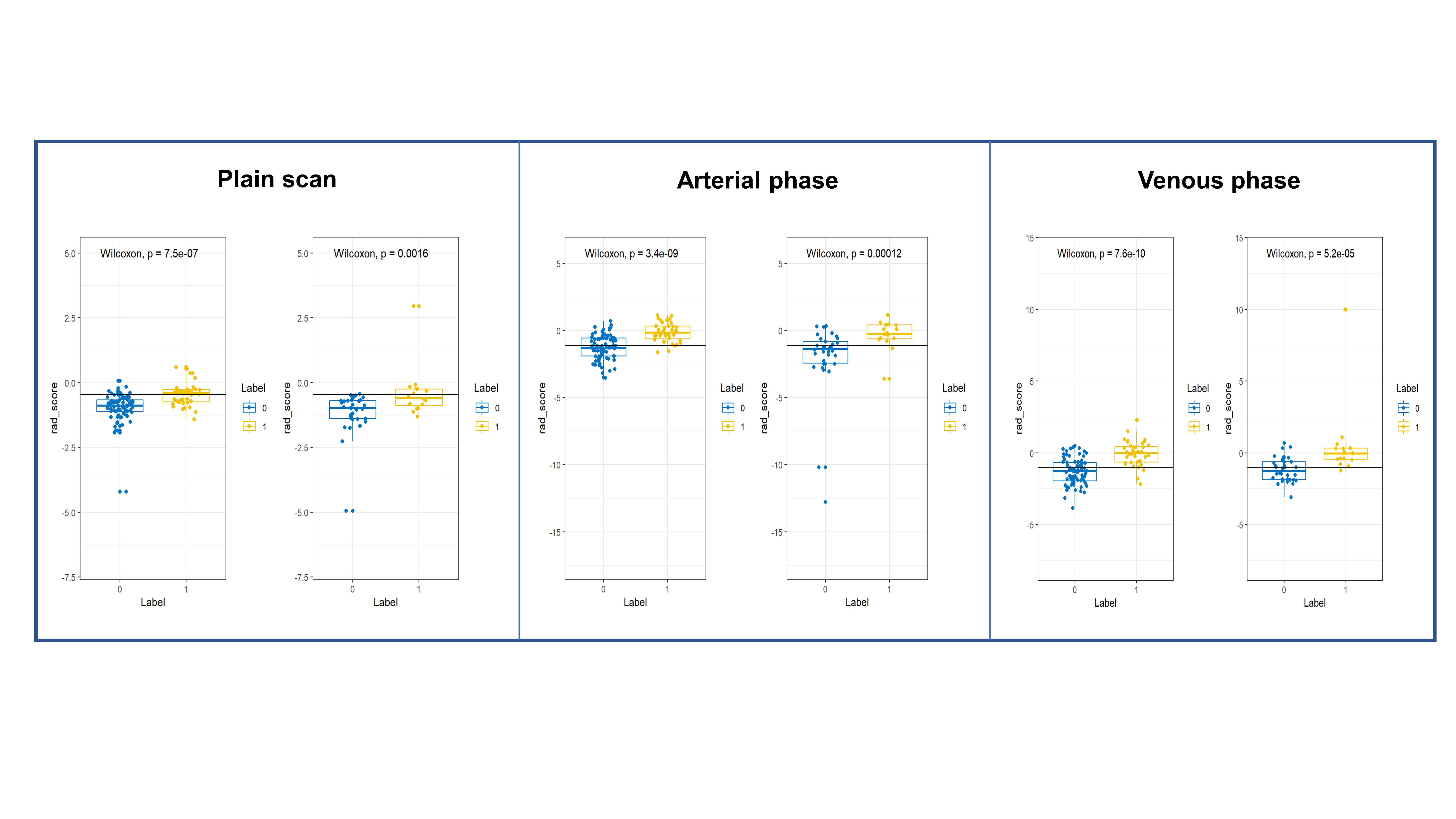


**III.** Two-phase radiomics model construction and performance evaluation

(1) The calculation formula of Radscores in two-phase radiomics model.

**Plain scan + Arterial phase radiomics model:**

***P+A-Radscore***= 0.569*AP_wavelet-LLL_glszm_GrayLevelNonUniformityNormalized +0.288*AP_log-sigma-1-0-mm-3D_glszm_SmallAreaEmphasis +0.01*AP_wavelet-LLL_glszm_LargeAreaLowGrayLevelEmphasis +0.306*AP_log-sigma-3-0-mm-3D_glszm_SizeZoneNonUniformityNormalized -0.39*PS_log-sigma-2-0-mm-3D_glcm_Idn+-0.164*AP_original_firstorder_Median -0.3*PS_wavelet-HHL_firstorder_Median +0.101*AP_wavelet-LLH_glszm_SizeZoneNonUniformityNormalized +0.045*PS_wavelet-HLH_gldm_LargeDependenceEmphasis +0.405*AP_original_firstorder_Kurtosis -0.186*AP_wavelet-LLL_glszm_LargeAreaHighGrayLevelEmphasis +0.004*AP_wavelet-LLL_glcm_InverseVariance -0.094*AP_wavelet-HHH_glszm_ZoneVariance -0.882

**Plain scan + Venous phase radiomics model:**

***P+V-Radscore***= 0.006*VP_wavelet-LLL_glszm_LargeAreaLowGrayLevelEmphasis -0.224*PS_log-sigma-2-0-mm-3D_firstorder_Maximum -0.528*VP_original_firstorder_Median -0.09*VP_wavelet-LLL_gldm_HighGrayLevelEmphasis +0.684*VP_wavelet-LLL_glcm_MaximumProbability -0.12*VP_log-sigma-5-0-mm-3D_glcm_Contrast +0.505*VP_log-sigma-1-0-mm-3D_glszm_SizeZoneNonUniformityNormalized +0.15*VP_log-sigma-3-0-mm-3D_glszm_SizeZoneNonUniformityNormalized -0.173*VP_wavelet-LLL_glszm_LargeAreaEmphasis -0.455*PS_log-sigma-2-0-mm-3D_glcm_Idn -0.189*VP_wavelet-LHH_glcm_Imc1 +0.204*VP_wavelet-LHH_firstorder_Mean -0.312*PS_wavelet-HHL_firstorder_Median -0.983

**Arterial phase + Venous phase radiomics model:**

***A+V-Radscore*** = -0.204*VP_original_firstorder_Median +0.251*AP_log-sigma-3-0-mm-3D_glszm_SizeZoneNonUniformityNormalized +0.252*AP_original_gldm_LowGrayLevelEmphasis +0.197*VP_wavelet-LHH_firstorder_Mean +0.24*VP_log-sigma-1-0-mm-3D_glszm_SizeZoneNonUniformityNormalized +0.065*VP_wavelet-HHH_firstorder_Median -0.226*AP_wavelet-LLL_gldm_DependenceEntropy -0.33*AP_wavelet-HLL_firstorder_InterquartileRange -0.232*AP_wavelet-LLL_glcm_JointAverage -0.199*AP_log-sigma-2-0-mm-3D_firstorder_InterquartileRange -0.928

1. **Fig. S5.** The comparison of two-phase radiomics models in ROC curves in the training cohort (A) and validation cohort (B).

**
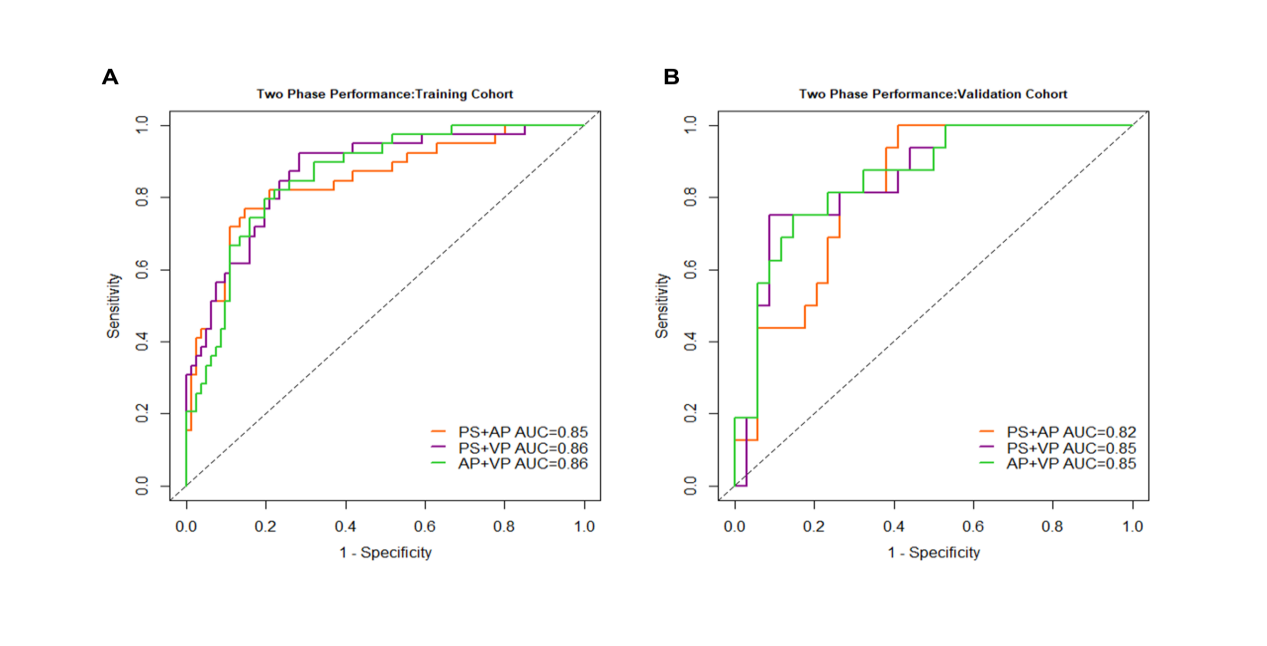
**

**IV.** Multi-phase radiomics model construction and calculation formulas of the MP-Radscore and combined nomogram.

1. The calculation formula of multi-phase Radscore and combined nomogram:

***MP-Radscore***= -0.52*AP_log-sigma-1-0-mm-3D_gldm_DependenceEntropy +0.015*AP_wavelet-LLL_glszm_LargeAreaLowGrayLevelEmphasis +0.303*VP_log-sigma-1-0-mm-3D_glszm_SizeZoneNonUniformityNormalized +0.352*VP_wavelet-LHH_firstorder_Mean -0.306*VP_original_firstorder_Median -0.166*VP_wavelet-LLL_glszm_LargeAreaEmphasis +0.163*AP_log-sigma-3-0-mm-3D_glszm_SizeZoneNonUniformityNormalized -0.741*AP_wavelet-LHL_glcm_Imc1 -0.293*VP_original_gldm_DependenceEntropy +0.33*PS_log-sigma-2-0-mm-3D_glszm_SmallAreaEmphasis -0.434*VP_wavelet-HHL_gldm_SmallDependenceLowGrayLevelEmphasis -0.641*PS_log-sigma-3-0-mm-3D_firstorder_Maximum +0.037*VP_wavelet-LLL_glszm_LowGrayLevelZoneEmphasis -0.079*VP_log-sigma-5-0-mm-3D_firstorder_InterquartileRange -1.067

***Nomoscor*e=** -0.307382965280555 + 0.52409593448835*Lesion Location - 0.268836657077142 *Cyst_number + MP-Radscore*1.44716439905756

1. **Fig. S6.** The calibration curves presented good consistency between the predictive and observation probabilities of the combined nomogram in the training cohort (A) and validation cohort (B).


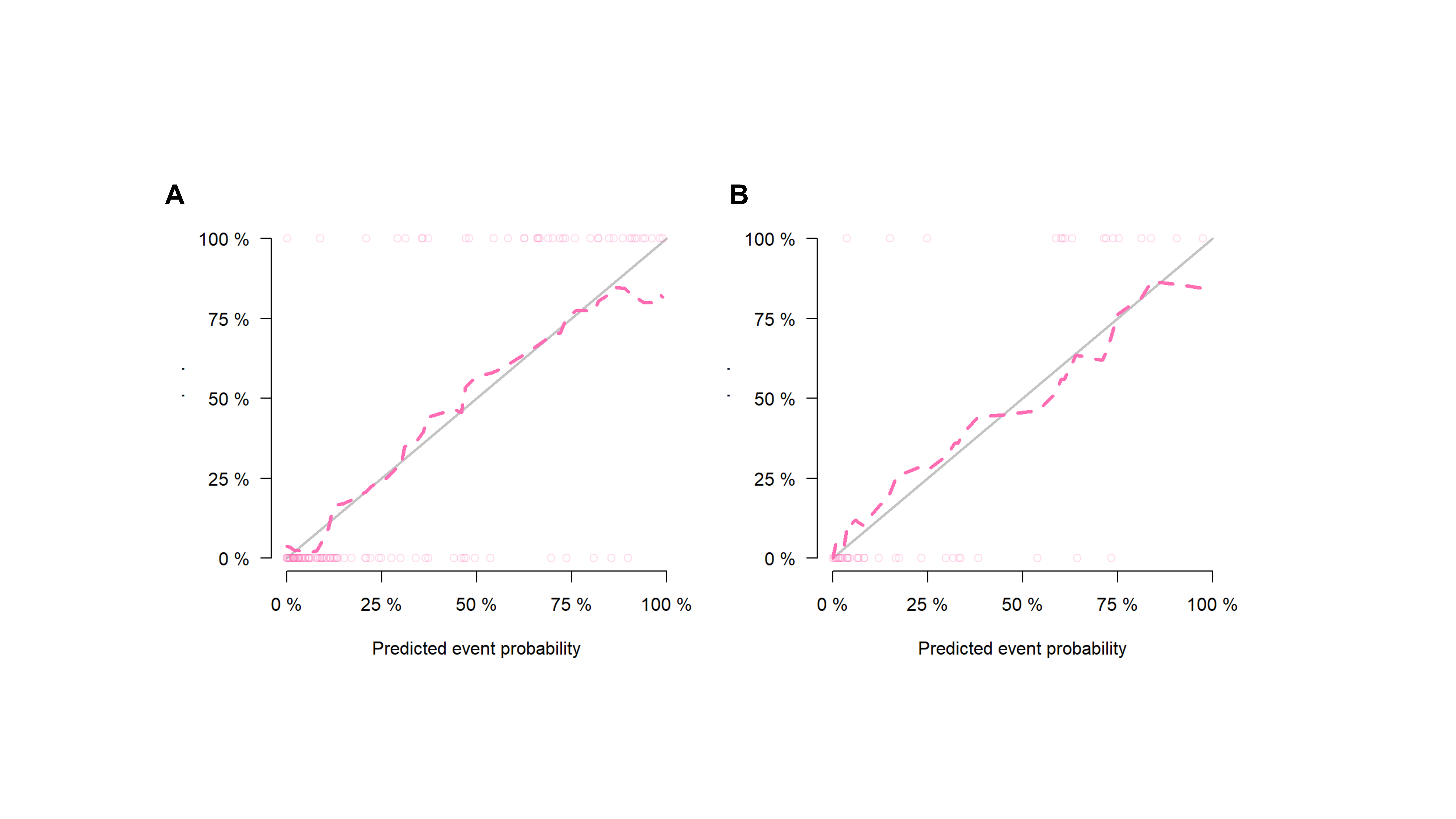


**V.** **R packages we used in this study**

The “glmnet” package was used for LASSO logistic regression. The “rms” package was used in the logistic regression analysis , calibration plots and VIF calculation. Calibration curves were established using bootstrapping validation with 1,000 resamples. ROC curves and Delong test were performed with the “pROC” package. The Hosmer-Lemeshow test was performed using the “generalhoslem” package. The “dca.R” package was used for decision curve analysis.

The relevant code used to build the model, which can be found on the website: https://github.com/jaho0528/radiomicspanc.
